# Retention rate of subcutaneous TNF inhibitors in axial spondyloarthritis in a multicentre study from the RIC-FRANCE network

**DOI:** 10.1038/s41598-024-52016-4

**Published:** 2024-01-16

**Authors:** Guillaume Larid, Guy Baudens, Georges Tiemdjo-Djimaffo, Pascal Coquerelle, Vincent Goeb, Marie Hélène Guyot, Laurent Marguerie, Frédéric Maury, Eric Veillard, Eric Houvenagel, Jean-Hugues Salmon, René-Marc Flipo, Elisabeth Gervais

**Affiliations:** 1 LITEC, Université de Poitiers, CHU Poitiers, 86000 Poitiers, France; 2 Private Practice, Valenciennes, France; 3 CHU Poitiers, Poitiers, France; 4 Bethune Hospital Center, Bethune, France; 5 University Hospital of Amiens-Picardie, Amiens, France; 6 Hôpital Victor Provo, Hospital of Roubaix, Roubaix, France; 7 Fondation Hopale, Berck-sur-Mer, France; 8 Private Practice, Beuvry, France; 9 Private Practice, Saint Malo, France; 10 Hôpital Saint Philibert, Hospital of Lomme, Lomme, France; 11 Centre Hospitalier Universitaire de Reims, Reims, France; 12 CHU Lille, Lille, France

**Keywords:** Rheumatology, Spondyloarthritis, Ankylosing spondylitis, Biological therapy

## Abstract

The objectives of our study were to assess retention rate, safety, and predictive factors for retention of subcutaneous (SC) TNF inhibitors (TNFi) (adalimumab (ADA), etanercept (ETN), golimumab (GOL), and certolizumab pegol (CZP)) in axial spondyloarthritis (axSpA) depending on the line of treatment in real-life conditions. A multicentre retrospective observational study was conducted including 552 patients fulfilling the ASAS criteria for axSpA followed in the RIC-France register who began SC-TNFi between 01/01/13 and 08/31/2018 for a total of 824 prescriptions. Taking all lines of treatment into account, GOL had a significantly higher retention rate compared with ADA, ETN, and CZP with a mean retention length of 59 months. As first-line bDMARDs, GOL had a significantly higher retention rate compared with ADA and ETN. ETN had the best retention rate when prescribed as at least 3rd bDMARD. Taking all lines of treatment into account, female sex, peripheral disease, BASDAI at initiation, and line of treatment were predictive factors for treatment cessation. Primary inefficiency was the most frequent reason for treatment cessation. In conclusion, GOL showed the highest retention rate in axSpA. Male sex, absence of peripheral disease, and early line of prescription were associated with better SC-TNFi retention in axSpA.

## Introduction

Spondyloarthritis (SpA) is a chronic inflammatory disease affecting both the peripheral and the axial skeleton. Axial spondyloarthritis (axSpA) encompasses both the radiographic (r-axSpA) and the non-radiographic (nr-axSpA) forms of the disease^1^.

Management of axSpA patients with persistently high disease activity despite NSAID use is based on bDMARDs, either TNFi or IL-17 inhibitors, as well as JAK inhibitors (upadacitinib and tofacitinib) with current practice and recommendations being to start with TNFi^2^. TNFi can be prescribed intravenously (IV) or subcutaneously (SC). Currently approved SC-TNFis for axSpA treatment are adalimumab (ADA), etanercept (ETN), certolizumab-pegol (CZP), golimumab (GOL) and, more recently in France, infliximab (IFX). In France, IL-17A inhibitors have been licensed since July 2016.

All of these treatments are considered as demonstrating comparable efficacy and clinical response rates^3^. That said, studies on retention of treatments in axSpA have yielded surprisingly divergent results^4–6^.

If a treatment is not considered sufficiently efficient, switching to another bDMARD is recommended^2^. Studies have shown that subsequent bDMARDs can be less effective than the previous ones^7,8^. However, literature is discordant, with some studies demonstrating similar retention, whatever the line of prescription^5,9^.

Concerning prognosis factors, male sex, obesity, comorbidities, line of prescription, and HLA-B27 have previously been among the factors identified, with divergent results^5,10–13^.

In this context of literature discrepancy, data from real-world studies can provide information for rheumatologists that is precious on account of its being closer to clinical practice. Their interest is supported by the need for constant integration of all available levels of evidence to ensure optimal quality of care^14^.

The objectives of our study were to assess retention rate and predictive factors for retention of subcutaneous SC-TNFi in axSpA depending on molecule and on line of treatment in real-life conditions. The safety of the different treatments was also analysed.

## Materials and methods

### Patients

The RIC-France Network is a database with shared informatic medical records of patients with chronic inflammatory arthritis, and it is used for clinical studies on rheumatic diseases^15,16^. Patients are included in the database and data are filled out by their rheumatologists during consultations. In the context of this study, data from each patient were completed based on their original medical records.

This is a retrospective, observational, multicentre study. We included patients fulfilling the ASAS criteria for axSpA^17^ followed in the multicentre RIC-France Network, and who began SC-TNFi between 01/01/13 and 08/31/2018. Follow-up started at the initiation of SC-TNFi and ended at the interruption date of treatment, death, or end of the study, whichever occurred first.

We did not include patients who started IV-TNFi, or began SC-TNFi outside of the inclusion period.

### Assessments

Patient characteristics were collected from their shared medical records: age, sex, HLA-B27 allele presence, disease duration, age at diagnosis, global pain evaluation using VAS (from 0 to 100), global physician opinion (from 0 to 10), presence of biological inflammation (using c-reactive protein or erythrocyte sedimentation rate), disease activity using BASDAI questionnaire (from 0 to 10). All the data on treatments received, therapeutic line, doses, treatment retention lengths, and reasons for treatment cessation have been collected.

X-Ray sacro-iliitis was defined using the New York criterion^18^. MRI sacro-iliitis was defined using the ASAS/OMERACT definition^19^.

Primary inefficiency was defined as an absence of response in the first 6 months following treatment initiation. Treatment cessation was defined as secondary inefficiency if loss of response occurred after 6 months of initial therapeutic response.

### Statistical analysis

Qualitative data were expressed as absolute numbers and percentages and quantitative data as median [25th percentile–75th percentile] since none of the quantitative data described were normally distributed according to Shapiro–Wilk test and Anderson–Darling tests. Univariable analysis was conducted using Chi^2^ (or Fisher exact test) for qualitative data. To compare treatment retention, a log-rank test using Kaplan Meier curves was used. In some analyses, median retention length was not calculable because the retention rate never dropped under 50% during the analysed period. For univariable and multivariable analysis of predictive factors of retention, a Cox proportional-hazards regression was performed. All variables with p < 0.15 in univariable analysis were included in the multivariable analysis. A p value at 0.05 was considered as significant. Statistical analysis was performed using GraphPad Prism (GraphPad Software, California) and MedCalc (MedCalc Software Ltd, Belgium).

### Ethics

The study was conducted in accordance with the Declaration of Helsinki. This study falls within the scope of the French Reference Methodology MR-004 according to 2016–41 law dated 26 January 2016 on the modernisation of the French health system. Our study involves the reuse of already recorded data, which require neither information, non-opposition of the included individuals or ethic committee approval.

## Results

### Patient characteristics and treatment prescription

Between 1st January 2013 and 31 August 2018, the records of 1081 patients with axSpA were included in the shared medical records database. A total of 552 patients were included in the study, representing 824 prescriptions. There were 418 first-line prescriptions, 230 second-line prescriptions, and 176 third-line prescriptions.

Principal characteristics of the patients are detailed in Table 1; 54.5% were male patients, median age was 44.0 years. Median disease duration was 95 months. Presence of HLA-B27 allele was screened in 470 patients and was positive in 345 patients (73.4%). Median BASDAI at initiation of treatment was 5.60 [4.35–6.65].

**Table 1:** Patient characteristics (n = 552).

Age (years; median [25–75 percentiles])	44.00 [36.00–52.00]
Male sex (n; %)	301 (54.5%)
BMI (kg/m^2^; median [25–75 percentiles])	25.25 [22.47–29.54]
Disease duration (months; median [25–75 percentiles])	95.00 [47.00–175.00]
HLA-B27 (n; %)	345/470 (73.4%)
Peripheral disease (n; %)	214/552 (38.8%)
Crohn’s disease (n; %)	34/473 (7.2%)
Ulcerative colitis (n; %)	17/470 (3.6%)
Uveitis (n; %)	94/479 (19.6%)
Psoriasis (n; %)	67/473 (14.2%)
X-Rays sacro-iliitis (n; %)	321/426 (75.4%)
MRI sacro-iliitis (n; %)	255/325 (78.5%)
X-Rays and MRI sacro-iliitis (n; %)	157/283 (55.5%)

Continuous values are shown as median [25th percentile–75th percentile] and categorical variables as absolute number and percentage; *SD* Standard deviation; *BMI* Body mass index; *MRI* Magnetic resonance imaging.

Prescription of each studied treatment is detailed in Table 2. ADA was the most widely prescribed as first and second-line treatment while GOL was the most prescribed in third-line. Only 7 patients had concomitant methotrexate prescription.

**Table 2:** Details of SC-TNFi prescription depending on the line of treatment (n; (%)).

	1st line	2nd line	At least 3rd line	Total
CZP	12 (2.87%)	21 (9.13%)	45 (25.56%)	78 (9.46%)
ADA	**160** (38.27%)	**107** (46.52%)	27 (15.34%)	294 (35.67%)
GOL	126 (30.14%)	41 (17.82%)	**79** (44.88%)	246 (29.85%)
ETN	120 (28.70%)	61 (26.52%)	25 (14.20%)	206 (25%)
Total	418 (50.7%)	230 (27.9%)	176 (21.4%)	824

Data are expressed as absolute number and percentage; CZP certolizumab pegol; *ADA* Adalimumab; *GOL* Golimumab; *ETN* etanercept.

Concerning first-line treatments, there was a statistically significant difference in prescription of each molecule (CZP vs ADA, ETN, or GOL, p < 0.0001; ADA vs GOL, p = 0.0132; ADA vs ETN, p = 0.0034) excepted between GOL and ETN (p = 0.6488).

Concerning second-line treatments, there was a statistically significant difference in prescription of each molecule (GOL vs ETN, p = 0.0248; GOL vs CZP, p = 0.0063; and p < 0.0001 for all other comparisons).

Concerning at least third-line treatments, there was a statistically significant difference in prescription of each molecule (GOL vs ADA, ETN, or CZP, p < 0.0001; CZP vs ADA, p = 0.0174; CZP vs ETN, p = 0.0076) except for ADA vs ETN (p = 0.7639).

Details of treatment prescription comparisons are found in Supplementary Table 1.

### Retention rates for treatments

#### Retention rates for the different treatments combined

**Figure 1:**
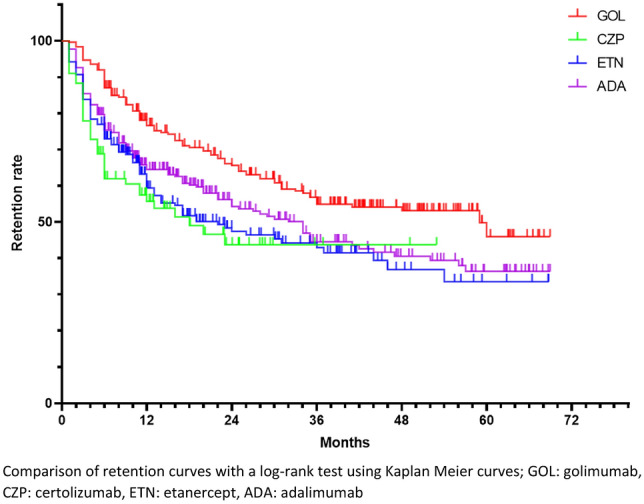
Retention rate of subcutaneous TNF inhibitors combining all lines of treatment. Comparison of retention curves with a log-rank test using Kaplan Meier curves; GOL: golimumab, CZP: certolizumab, ETN: etanercept, ADA: adalimumab.

Retention curves for each treatment are represented in Fig. 1 (n = 824).

Median retention length was 59 months for GOL, 34 months for ADA, 22 months for ETN, and 18 months for CZP.

GOL had a significantly higher retention rate compared with ADA (p = 0.002), ETN (p < 0.0001), and CZP (p = 0.0001). Other comparisons were not significant.

#### Retention rates in first-line treatment

**Figure 2:**
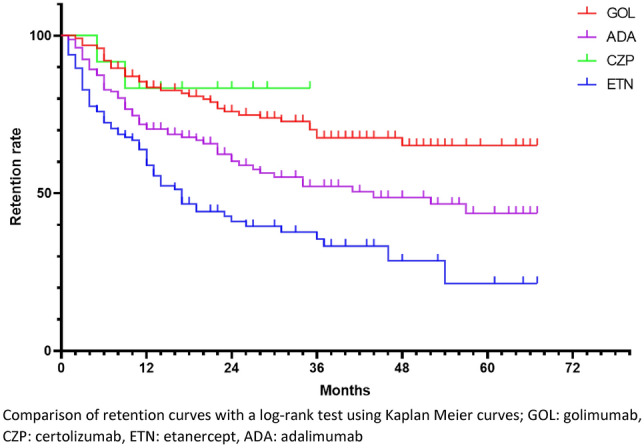
Retention rate of subcutaneous TNF inhibitors prescribed as first-line bDMARDs. Comparison of retention curves with a log-rank test using Kaplan Meier curves; GOL: golimumab, CZP: certolizumab, ETN: etanercept, ADA: adalimumab.

All in all, 418 first-line bDMARD prescriptions were studied. Retention curves for each treatment are represented in Fig. 2.

Median retention length was not calculable for GOL and CZP because of a retention rate never falling under 50% during the analysed period. Median retention length was 44 months for ADA, and 17 months for ETN.

GOL had a significantly higher retention rate compared to ADA (p = 0.0025) and ETN (p < 0.0001). ADA had a higher retention rate compared to ETN (p = 0.0015). ETN had a significantly lower retention rate compared to CZP (p = 0.0349). The other comparisons were not significant.

Retention rates at 12 and 24 months are detailed in Fig. 5.

At 12 months, GOL retention rate was significantly higher compared to ADA (p = 0.0166) and ETN (p < 0.0001). Retention rate for ADA was significantly higher than for ETN (p = 0.0292). The other comparisons were not significant.

At 24 months, GOL retention rate was significantly higher compared to ADA (p = 0.0287) and ETN (p < 0.0001). Retention rate for ADA was significantly higher than for ETN (p = 0.0004). ETN retention rate was significantly lower than for CZP (p = 0.0132). The other comparisons were not significant.

#### Retention rates in second-line treatment

**Figure 3:**
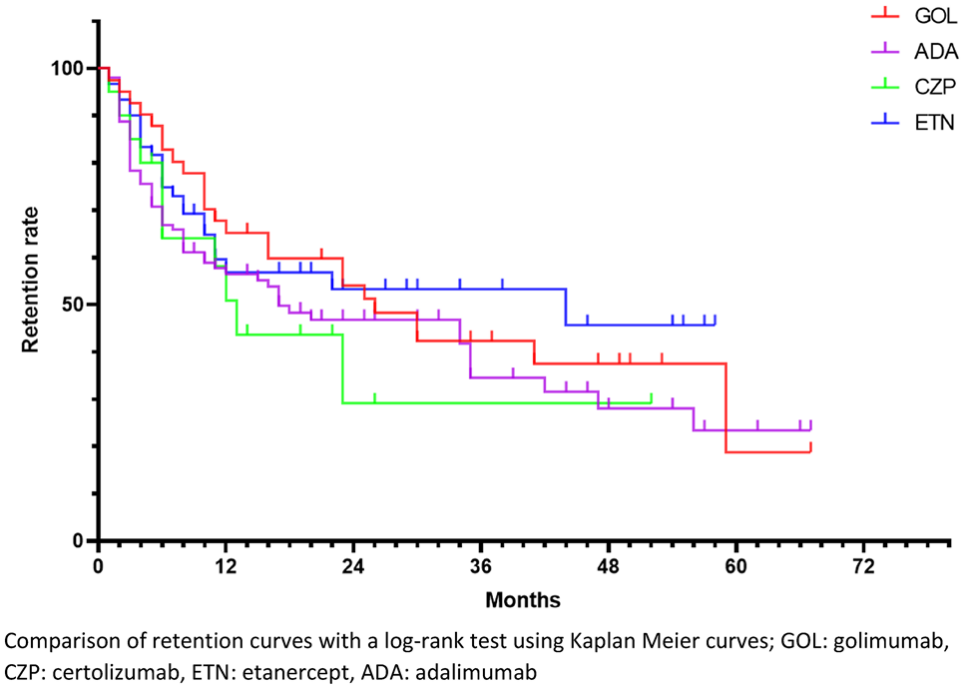
Retention rate of subcutaneous TNF inhibitors prescribed as second-line bDMARDs. Comparison of retention curves with a log-rank test using Kaplan Meier curves; GOL: golimumab, CZP: certolizumab, ETN: etanercept, ADA: adalimumab.

All in all, 230 second-line bDMARD prescriptions were studied. Retention curves for each treatment are represented in Fig. 3.

Median retention length was 57 months for ADA, 26 months for GOL, 13 months for CZP, and 44 months for ETN.

None of the comparisons of retention rates between treatments were significant.

Retention rates at 12 and 24 months are detailed in Fig. 5. There were no statistical differences between treatments at the different time points.

#### Retention rates in third-line or more treatment

**Figure 4:**
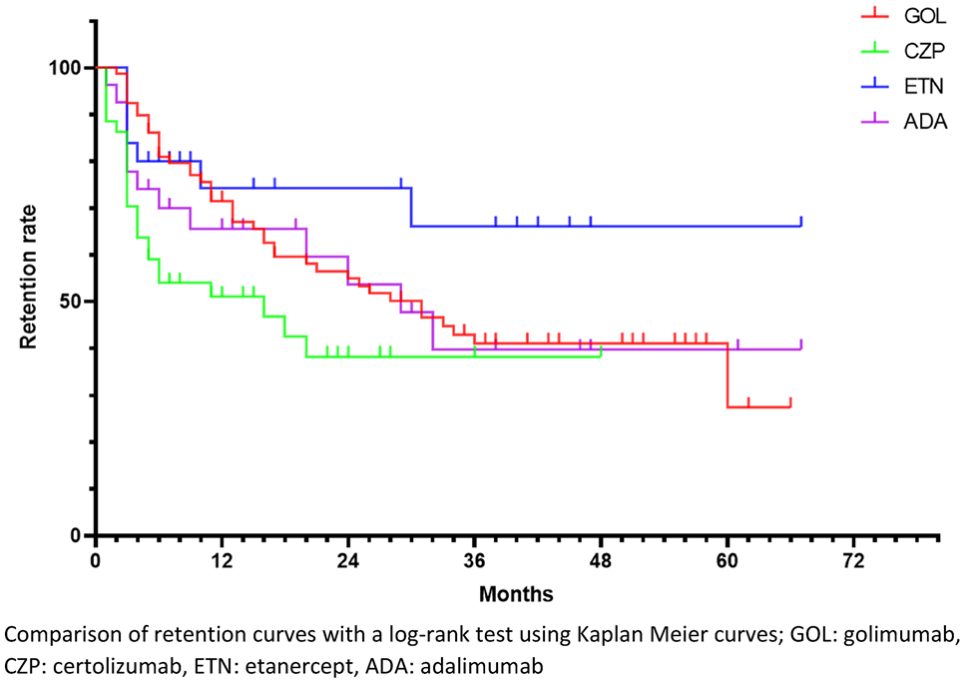
Retention rate of subcutaneous TNF inhibitors prescribed as third-line bDMARDs. Comparison of retention curves with a log-rank test using Kaplan Meier curves; GOL: golimumab, CZP: certolizumab, ETN: etanercept, ADA: adalimumab.

All in all, 176 third-line bDMARDs prescription were studied. The retention curves for each treatment are represented in Fig. 4.

Median retention length was not calculable for ETN, because retention rate was always over 50% during the analysed period. Median retention length was 29 months for ADA, 31 months for GOL, and 16 months for CZP.

CZP had a significantly lower retention rate compared to ETN (p = 0.0208) and GOL (p = 0.0262). The other comparisons were not significant.

**Figure 5:**
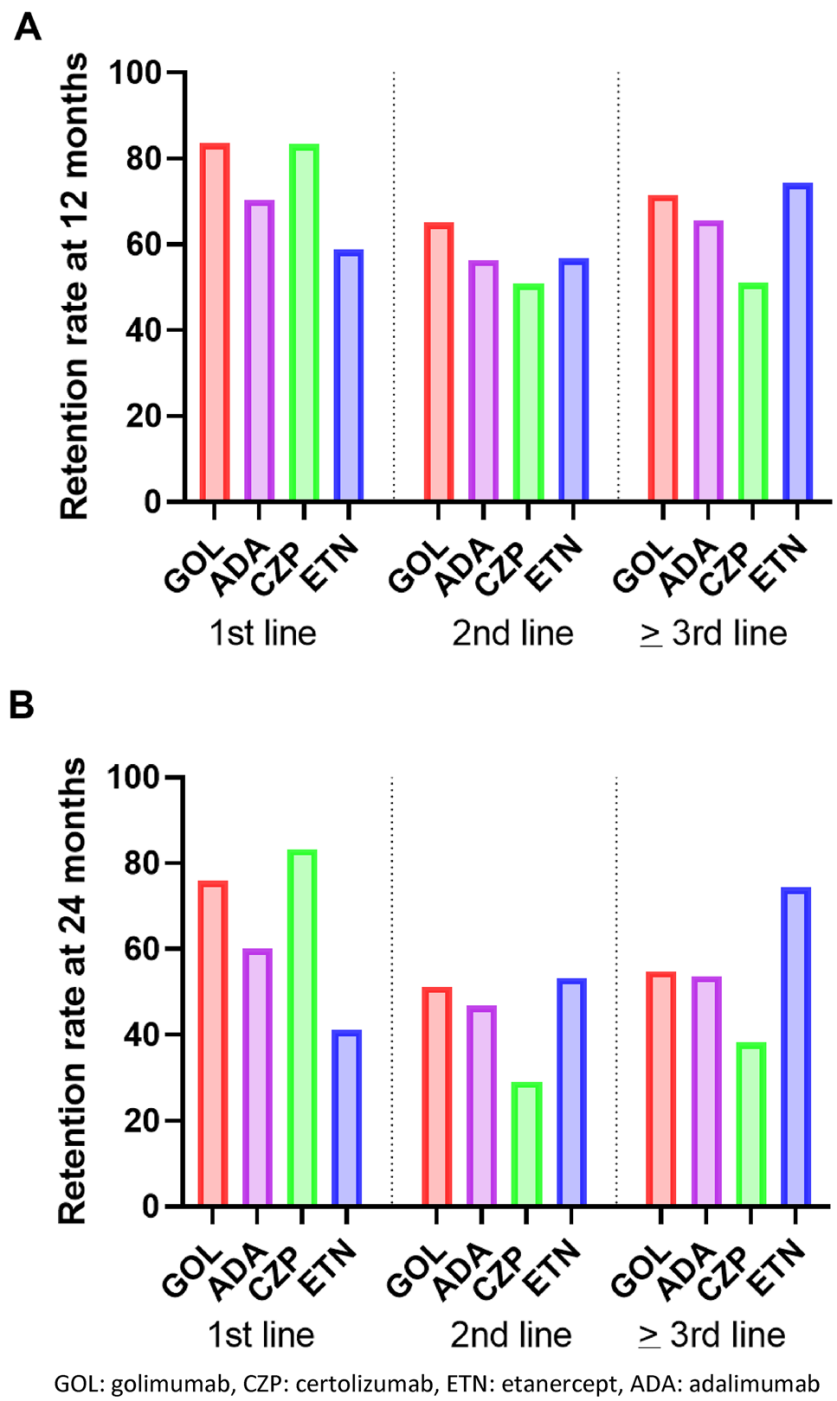
Retention rate of subcutaneous TNF inhibitors at 12 (**A**) and 24 months (**B**) depending on the bDMARD line of treatment. GOL: golimumab, CZP: certolizumab, ETN: etanercept, ADA: adalimumab.

Retention rates at 12 and 24 months are detailed in Fig. 5.

At 12 months, the ETN retention rate was significantly higher compared to CZP (p = 0.0417). GOL retention rate was higher compared to ADA (p = 0.0261) and to CZP (p < 0.0001). The other comparisons were not significant.

At 24 months, the ETN retention rate was significantly higher compared to CZP (p = 0.0409). GOL retention rate was higher compared to CZP (p = 0.0047). The other comparisons were not significant.

Given that there appeared to be visually better retention in third-line than in second-line treatment, retention rates for each of the second and third-line treatments were compared. There were no significant differences in retention between prescription in second or third-line treatments (p = 0.266 for ETN, p = 0.721 for GOL, p = 0.444 for ADA, and p = 0.7706 for CZP).

#### Retention of all SC-TNFis depending on the line of treatment, sex of patients, and HLA-B27 status

Comparisons of the respective retention rates of SC-TNFis of each line showed higher median retention length in first- line compared with second and at least third-line with medians of 48 months, 23 months, and 29 months respectively (p = 0.0004) (Supplementary Fig. 1). At 12 months, retention rates were 71.6% for 1st line treatment, 57.8% for 2nd line treatment and 65.7% for third-line treatment (1st line vs 2nd line: p < 0.0001; 1st line vs at least 3rd line and more: p = 0.019; 2nd line vs at least 3rd line: p = 0.074). At 24 months, survival was 60.5% for 1st line treatment, 48.2% for 2nd line treatment and 54.7% for at least third line treatment (1st line vs 2nd line: p = 0.009; 1st line vs at least 3rd line: p = 0.60; 2nd line vs at least 3rd line: p = 0.086).

Comparisons of retention rate of SC-TNFis depending on the sex showed higher median retention length in men with a median of 54 months vs 25 months in women (p = 0.0004) (Supplementary Fig. 2). At 12 months, retention rate was 61.1% for females and 71.6% for males (p = 0.019). At 24 months, retention rate was 50.5% for female and 60.3% for male patients (p = 0.009). Comparisons of retention rates of ETN and monoclonal antibodies (MAb) in men showed better retention of MAb (p = 0.0376) with median retention length of 57 months vs 36 months for ETN. In women, median retention length was 27 months for MAb and 17 months for ETN without significant differences (p = 0.2315) (Supplementary Fig. 3).

Comparisons of retention rate of SC-TNFis depending on HLA-B27 positivity showed higher median retention length in HLA-B27 + patients with a median of 36 months vs 22 months in HLA-B27- (p = 0.0139) (Supplementary Fig. 4).

### Reasons for treatment interruption

Out of the 824 prescriptions studied, 385 were interrupted. Primary inefficiency was the most frequent reason for treatment cessation (136 prescriptions, 35.32%). Other reasons were secondary inefficiency (122 prescriptions, 31.69%), side effects (83 prescriptions, 21.56%), and others (33 prescriptions, 8.57%). Reason for treatment cessation was not reported in 11 cases (2.86%).

Reported side effects were 3 cancers (3.6%) (2 with GOL, 1 with ADA), asthenia for 4 patients (4.8%), cutaneous side-effects for 23 patients (27.7%), moderate neutropenia for 1 patient (1.2%), hypertension for 2 patients (2.4%), digestive intolerance for 4 patients (4.8%), recurrent infections for 13 patients (15.7%), and various reasons (including pregnancy or programmed surgery, for example) for 33 patients (39.8%).

### Predictive factors of treatment cessation

When all treatments are considered (Table 3), female sex (p = 0.0006), absence of HLA-B27 (p = 0.0158), peripheral disease (p < 0.0001), normal SI X-rays (p = 0.0085), higher BASDAI at initiation (p < 0.0001) and line of treatment (p = 0.0040) were significant predictive factors for treatment cessation in univariable analysis, while treatment by GOL was a predictive factor for treatment retention (vs ADA as reference; p = 0.0048). Female sex (p = 0.0357), peripheral disease (p = 0.0452), higher BASDAI at initiation (p = 0.0161), line of treatment (p = 0.0442) were significant predictive factors for cessation in multivariable analysis and treatment by GOL (vs ADA as reference; p = 0.0349) was a significant predictive factor for retention.

**Table 3:** Predictive factors for retention of treatments (Cox proportional-hazards regression analysis).

	Univariable analysis		Multivariate analysis	
	Hazard Ratio (CI 95%)	p	Hazard Ratio (CI 95%)	P
Female sex	1.4306 (1.1674–1.7532)	***0.0006***	1.4317 (1.0243–2.0011)	**0.0357**
B27 negativity	1.3329 (1.0554 – 1.6834)	***0.0158***	1.1368 (0.7915–1.6326)	0.4876
Peripheral disease	1.5905 (1.2988–1.9469)	** < *0.0001***	1.3954 (1.0072–1.9333)	**0.0452**
Crohn’s disease	1.0002 (0.6650–1.5044)	0.9993	–	–
Ulcerative colitis	0.9387 (0.5274–1.6707)	0.8297	–	–
Uveitis	0.8762 (0.6698–1.1461)	0.3347	–	–
Psoriasis	1.1542 (0.8620–1.5424)	0.3355	–	–
Normal SI X-Rays	1.3896 (1.0875–1.7755)	***0.0085***	0.7500 (0.5019–1.1209)	0.1606
Normal SI MRI	1.1299 (0.8391–1.5214)	0.4211	–	–
Age	0.9969 (0.9883–1.056)	0.4859	–	–
Disease duration	0.9990 (0.9979–1.0002)	*0.0907*	0.9988 (0.9969–1.0007)	0.2162
BMI	1.0083 (0.9771–1.0404)	0.6077	–	–
BASDAI at initiation	1.1899 (1.0471–1.2906)	** < *0.0001***	1.1259 (1.0222–1.2401)	**0.0161**
Line of treatment	1.1958 (1.0588–1.3506)	***0.0040***	1.2432 (1.0057–1.5370)	**0.0442**
Treatments				
ADA	REFERENCE	–	REFERENCE	–
CZP	1.3456 (0.9347–1.9373)	*0.1103*	1.1865 (0.6691–2.1041)	0.5595
ETN	1.1416 (0.8815–1.4784)	0.3153	1.0309 (0.6819–1.5585)	0.8853
GOL	0.6908 (0.5343–0.8931)	**0.0048**	0.6445 (0.4285–0.9694)	**0.0349**

For the analysis of predictive factors of retention, a Cox proportional-hazards regression model was employed. All variables with p < 0.15 in univariable analysis were included in the multivariable analysis; *Variables in italics are included in the multivariable analysis (p* < *0.15): sex, B27 status, peripheral disease, SI X-rays, disease duration, BASDAI at initiation, line of treatment, type of treatment*; Significant values are in bold (p < 0.05); *BMI* Body mass index; *MRI* Magnetic resonance imaging; *SI* Sacro-iliac; *CZP* Certolizumab pegol; *ADA* Adalimumab; *GOL* Golimumab; *ETN* Etanercept; *CI* Confidence interval.

Considering predictive factors for each treatment (Table 4), significant predictive factors for GOL cessation were female sex (p = 0.0248), peripheral disease (p = 0.0424), and line of treatment (p = 0.0002) in univariable analysis. None of these factors were significant in multivariable analysis.

**Table 4:** Predictive factors of treatment cessation for each SC-TNFi.

	GOL				ADA				ETN				CZP			
	Univariable analysis		Multivariable analysis		Univariable analysis		Multivariable analysis		Univariable analysis		Multivariable analysis		Univariable analysis		Multivariable analysis	
	Hazard Ratio (CI 95%)	p	Hazard Ratio (CI 95%)	p	Hazard Ratio (CI 95%)	p	Hazard Ratio (CI 95%)	p	Hazard Ratio (CI 95%)	p	Hazard Ratio (CI 95%)	p	Hazard Ratio (CI95%)	p	Hazard Ratio (CI 95%)	p
Female sex	1.5612 (1.0581–2.3036)	***0.0248***	1.6435 (0.8323–3.0272)	0.1109	1.7889 (1.2721–2.5156)	***0.0008***	2.8420 (1.0832–7.45700)	**0.0338**	1.1958 (0.8058–1.7746)	0.3746	–	–	0.5587 (0.2920–1.0691)	*0.0787*	0.6717 (0.3405–1.3248)	0.2508
B27 presence	0.6923 (0.4337–1.1051)	*0.1233*	0.7729 (0.4019–1.4863)	0.4400	0.6615 (0.4523–0.9675)	***0.0331***	0.8878 (0.3795–2.0768)	0.7837	0.8921 (0.5740–1.3865)	0.6118	–	–	1.4593 (0.6054–3.5176)	0.3998	–	–
Peripheral disease	1.5056 (1.0142–2.2350)	***0.0424***	1.2319 (0.6291–2.4120)	0.5430	1.5877 (1.1366–2.2179)	***0.0067***	2.3822 (0.9116–6.2253)	0.0765	1.4301 (0.9604–2.1295)	*0.0782*	1.9074 (0.8815–4.1268)	0.1010	1.6917 (0.8864–3.2289)	*0.1109*	1.5150 (0.7773–2.9529)	0.2225
Crohn’s disease	0.7366 (0.2979–1.8210)	0.5079	–	–	0.8671 (0.4775–1.5747)	0.6395	–	–	1.4564 (0.5305–3.9978)	0.4656	–	–	2.0639 (0.7238–5.8855)	0.1753	–	–
Ulcerative colitis	0.7640 (0.2415–2.4167)	0.6468	–	–	0.9826 (0.4577–2.1096)	0.9641	–	–	1.0507 (0.1454–7.5912)	0.9609	–	–	4.5502 (0.5981–31.6158)	*0.1433*	2.3360 (0.2918–18.7020)	0.4241
Uveitis	1.2624 (0.7905–2.0162)	0.3293	–	–	0.7099 (0.4504–1.1189)	*0.1399*	0.8722 (0.2387–3.1867)	0.8362	0.7332 (0.3880–1.3855)	0.3391	–	–	0.8666 (0.4065–1.8477)	0.7109	–	–
Psoriasis	1.2048 (0.6899–2.1040)	0.5124	–	–	1.2302 (0.7531–2.0095)	0.4080	–	–	0.9810 (0.5528–1.7412)	0.9479	–	–	1.1707 (0.4850–2.8261)	0.7259	–	–
Positive SI X-Rays	0.6304 (0.3745–1.0614)	*0.0826*	1.2407 (0.5112–3.0114)	0.6336	0.8306 (0.5601–1.2316)	0.3557	–	–	0.7179 (0.4521–1.1401)	0.1602	–	–	0.9799 (0.4410–2.1770)	0.9602	–	–
Positive SI MRI	0.8294 (0.4497–1.5297)	0.5493	–	–	0.6491 (0.3960–1.0639)	*0.0865*	0.6590 (0.2273–1.9108)	0.4426	1.0694 (0.6212–1.8412)	0.8086	–	–	2.0173 (0.7603–5.3527)	0.1587	–	–
Age	0.9966 (0.9811–1.0124)	0.6724	–	–	0.9986 (0.9844–1.0131)	0.8509	–	–	0.9943 (0.9768–1.0122)	0.5302	–	–	0.9990 (0.9683–1.0305)	0.9477	–	–
Disease duration	0.9999 (0.9977–1.0021)	0.9382	–	–	0.9989 (0.9969–1.0009)	0.2657	–	–	0.9978 (0.9956–1.0001)	*0.0590*	0.9978 (0.9944–1.0011)	0.1955	1.0016 (0.9982–1.0051)	0.3517	–	–
BMI	1.0456 (0.9810–1.1144)	0.1704	–	–	0.9470 (0.89226–1.0048)	*0.0717*	0.9300 (0.8517–1.0156)	0.1062	1.0374 (0.9931–1.0837)	*0.0992*	1.0184 (0.9635–1.0765)	0.5187	0.9558 (0.6824–1.3388)	0.7926	–	–
BASDAI at initiation	1.1206 (0.9640–1.3026)	*0.1383*	1.1029 (0.9212–1.3205)	0.2861	1.1658 (0.991–1.3604)	*0.0514*	1.0143 (0.7287–1.4118)	0.9330	1.2438 (1.0666–1.4505)	*0.0054*	1.6648 (1.12265–2.2595)	**0.0011**	1.1718 (0.9355–1.4677)	0.1676	–	–
Line of treatment	1.4970 (1.2106–1.8550)	**0.0002**	1.3589 (0.9659–1.9117)	0.0782	1.3113 (1.0348–1.6618)	***0.0249***	0.0000 (9.36E^−191^-184E^+177^)	0.9545	0.6953 (0.5081–0.9514)	**0.0231**	0.5011 (0.1277–1.9661)	0.3218	0.6731 (1.0251–2.7307)	***0.0395***	1.7366 (1.0358–2.9115)	**0.0363**

For the analysis of predictive factors of retention, a Cox proportional-hazards regression was performed. All variables with p < 0.15 in univariable analysis were included in the multivariable analysis; *Variables in italics are included in the multivariable analysis (p* < *0.15)*; Significant values are in bold (p < 0.05); *BMI* Body mass index; *MRI* Magnetic resonance imaging; *SI* Sacro-iliac; *CZP* Certolizumab pegol; *ADA* Adalimumab; *GOL* Golimumab; *ETN* Etanercept; *CI* Confidence interval.

For ADA, significant predictive factors for cessation were female sex (p = 0.0008), peripheral disease (p = 0.0067), and line of treatment (p = 0.0249) in univariable analysis, while HLA-B27 positivity was associated with better treatment retention (p = 0.0331). Only female sex was associated with treatment cessation in multivariable analysis (p = 0.0338).

For ETN, significant predictive factors for cessation were higher BASDAI at initiation (p = 0.0054) and line of treatment (p = 0.0231) in univariable analysis. Only high BASDAI at initiation was associated with treatment cessation in multivariable analysis (p = 0.0043).

For CZP, the only significant predictive factor for retention was early-line prescription of treatment in both univariable (p = 0.0395) and multivariable analysis (p = 0.0363).

Considering predictive factors for each line of treatment (Table 5), significant predictive factors for first-line treatment cessation in univariable analysis were female sex (p = 0.0001), HLA-B27 absence (p = 0.0016), presence of peripheral disease (p < 0.0001), absence of X-ray sacro-iliitis (p = 0.0009), absence of MRI sacro-iliitis (p = 0.0379), and higher BASDAI at initiation (p < 0.0021). Taking ADA as reference treatment, ETN was more likely to be interrupted (p = 0.0021), while GOL was more likely to be maintained (p = 0.0036). In multivariable analysis, only female sex (p = 0.0103) and peripheral disease remained significant (p = 0.0004).

**Table 5:** Predictive factors of treatment cessation for each line of treatment.

	1st line				2nd line				3rd line and more			
	Univariable analysis		Multivariable analysis		Univariable analysis		Multivariable analysis		Univariable analysis		Multivariable analysis	
	Hazard Ratio (CI 95%)	p	Hazard Ratio (CI 95%)	p	Hazard Ratio (CI 95%)	p	Hazard Ratio (CI 95%)	p	Hazard Ratio (CI 95%)	p	Hazard Ratio (CI 95%)	p
Female sex	1.8477 (1.3630–2.5049)	***0.0001***	2.1653 (1.2004–3.9058)	**0.0103**	1.4611 (1.0135–2.1063)	***0.0422***	1.4782 (0.8278–2.6395)	0.1864	0.7646 (0.4996–1.1700)	0.2162	–	–
B27 presence	0.5856 (0.4202–0.8160)	***0.0016***	0.9728 (0.5562–1.7014)	0.9229	0.8729 (0.5788–1.3165)	0.5167	–	–	1.1370 (0.6336–2.0404)	0.6670	–	–
Peripheral disease	1.8949 (1.4024–2.5604)	** < *0.0001***	2.7860 (1.5775–4.9309)	**0.0004**	1.3938 (0.9717–1.9994)	*0.0713*	0.9613 (0.5423–1.7042)	0.8926	1.1656 (0.7637–1.7791)	0.4775	–	–
Crohn’s disease	0.6066 (0.2678–1.3741)	0.2309	–	–	1.2511 (0.6514–2.4032)	0.5011	–	–	1.2018 (0.5988–2.4124)	0.6050	–	–
Ulcerative colitis	1.0590 (0.4952–2.2647)	0.8825	–	–	0.6126 (0.1943–1.9316)	0.4029	–	–	1.7603 (0.4269–7.2578)	0.4340	–	–
Uveitis	0.6708 (0.4293–1.0481)	*0.0795*	0.7456 (0.2904–1.9141)	0.5417	0.7758 (0.4812–1.2510)	0.2977	–	–	1.4003 (0.8568–2.2887)	0.1792	–	–
Psoriasis	1.3682 (0.8695–2.1531)	0.1753	–	–	1.0501 (0.6155–1.7917)	0.8576	–	–	0.9162 (0.5274–1.5917)	0.7561	–	–
Positive SI X-Rays	0.5516 (0.3878–0.7846)	***0.0009***	1.2190 (0.6416–2.3163)	0.5453	0.8644 (0.5581–1.3387)	0.5138	–	–	1.0530 (0.6043–1.8349)	0.8554	–	–
Positive SI MRI	0.6422 (0.4228–0.9756)	***0.0379***	0.6371 (0.3295–1.2318)	0.1801	1.1586 (0.6882–1.9504)	0.5797	–	–	1.5062 (0.7095–3.1975)	0.2863	–	–
Age	1.0048 (0.9923–1.0174)	0.4528	–	–	0.9802 (0.9643–0.9962)	***0.0157***	0.9790 (0.9521–1.0066)	0.1352	0.9939 (0.9754–1.0128)	0.5274	–	–
Disease duration	0.9986 (0.9966–1.0005)	*0.1395*	0.9970 (0.9934–1.0005)	0.0912	0.9970 (0.9949–0.9991)	***0.0056***	0.9993 (0.9962–1.0024)	0.6711	1.0009 (0.9985–1.0034)	0.4596	–	–
BMI	1.0103 (0.9789–1.0427)	0.5254	–	–	0.8318 (0.5003–1.3832)	0.4779	–	–	0.9907 (0.9490–1.0342)	0.6690	–	–
BASDAI at initiation	1.3519(1.1801–1.5487)	** < *0.0001***	1.1847 (0.9945–1.4113)	0.0577	1.1204 (0.9779–1.2838)	*0.1015*	1.0471 (0.9046–1.2120)	0.5378	1.0712 (0.9297–1.2341)	0.3414	–	–
Treatments							–	–			–	–
ADA	REFERENCE	–	REFERENCE	–	REFERENCE	–			REFERENCE	–		
CZP	0.4194(0.1026–1.7148)	0.2265	1.1857(0.2642–5.3204)	0.8240	1.0992 (0.5766–2.0956)	0.7738			1.6017 (0.8101–3.1666)	0.1756		
ETN	1.7129(1.2162–2.4127)	***0.0021***	1.3759(0.7368–2.5694)	0.3166	0.7424(0.4652–1.1848)	0.2117			0.5375(0.2143–1.3483)	0.1858		
GOL	0.5451 (0.3622–0.8204)	***0.0036***	0.9947 (0.4739–2.0879)	0.9947	0.7867 (0.4891–1.2654)	0.3226			0.9380 (0.5028–1.7496)	0.8405		

For the analysis of predictive factors of retention, a Cox proportional–hazards regression was performed. All variables with p < 0.15 in univariable analysis were included in the multivariable analysis; *Variables in italics are included in the multivariable analysis (p* < *0.15)*; Significant values are in bold (p < 0.05); *BMI* Body mass index; *MRI* Magnetic resonance imaging; *SI* Sacro-iliac; *CZP* Certolizumab pegol; *ADA* Adalimumab; *GOL* Golimumab; *ETN* Etanercept; *CI* Confidence interval.

For second-line treatments, female sex (p = 0.0422), lower age at initiation (p = 0.0157) and shorter disease duration (p = 0.0056) were significant predictors for treatment cessation in univariable analysis. None of these factors were significant in multivariable analysis.

For at least third-line treatments, no predictive factors for treatment cessation were identified in either univariable or multivariable analysis.

## Discussion

Our study reports the results of an analysis of 824 prescriptions of SC-TNFi for axSpA in real-life conditions. These prescriptions were made both by independent rheumatologists and hospital rheumatologists, which is the strength of our study since it guarantees representativeness of our population for daily practice.

The main result is that, among all therapeutic lines, GOL had the best retention rate of all SC-TNFis in axial spondyloarthritis. GOL was previously reported as a well-maintained bDMARD in axSpA, with higher retention rates compared with rheumatoid arthritis ^20,21^. A recent systematic literature review focusing on GOL found that this treatment may have higher persistence than other TNFi ^4^. Persistence of GOL in axSpA was studied in a recent post-hoc analysis of the GO-PRACTICE trial with persistence of 52.6% at 24 months, which is more than 10% lower than in our study ^22^. In accordance with GO-PRACTICE, a recent review of the literature showed a GOL retention rate of 55.4% at 1 year and 43% at 2 years ^23^. Similarly, median discontinuation time reported for GOL in axSpA by Rahman and al. was 33.6 months vs 59 months in our study^24^. Nevertheless, retention rates of GOL in our study were concordant with previous reports^20^ when prescribed as 1st line bDMARDs, while retention rates in our study were lower when prescribed as 2nd line bDMARDs^25^. Reasons for better GOL persistence are numerous and depend on each patient. Previous studies have identified monthly injection rhythm^26,27^ as a factor influencing bDMARD retention. Moreover, it is known that patient satisfaction with SC-TNFi has an impact on treatment persistence, which has been studied with GOL auto injector ^26^.

More generally, whether a particular SC-TNFi has better retention in axSpA is still an unresolved question, and the literature provides diverging results. Indeed, previous studies had reported an absence of difference between TNFi in axSpA ^5,9,12,28–32^ while others had, like ours, shown retention differences between the different molecules whether in terms of retention rates or retention length.

When considering retention rates in our study in comparison with other studies, the results are discordant. At one year, Heiberg et al. reported a retention rate of 75.4% for ETN and 71.4% for ADA, which is similar to our results for ADA, but far more for ETN than in our study ^33^. Concordantly, Brocq et al. found retention rates of 76% at 12 months for ETN, whatever the line ^34^. Retention rates of treatments at two years were 55% in our study, which is lower than previously reported rates of up to 74% ^5^. In our study, retention rate of treatments at three years was 47%, while retention rates of 63% and 76% were reported in the literature in axSpA patients ^6,35^. After 3 years, retention rates higher than 78% were reported for ADA and ETN ^29^.

In our study, line of prescription of SC-TNFi influenced retention rates. Indeed, the retention rate of SC-TNFi as first-line bDMARDs in axSpA was higher compared with further therapeutic lines as previously reported ^30,36^. Median retention length of 48 months for 1st line TNFi was concordant with the results in a Korean report ^37^. Mean retention length of second-line TNFi was 23 months, which is higher than previously reported duration ^38^ but concordant with other reports ^39^. Other studies did not find such comparable influence of the line of prescription ^40^. In the Rosales-Alexander et al. study, mean retention rates of treatments were higher for all therapeutic lines.

Line of prescription also influences retention rate and length of each molecule differentially. In our study, as first-line bDMARDs, GOL and CZP had the best retention rates while ETN had the least retention. Early-line prescription was also a predictive factor for CZP treatment retention in the multivariable analysis. This is discordant with a previously published study, in which SpA patients showed similar retention rates of CZP, regardless of the line of treatment ^41^. It is important to notice that only a few patients in our study were treated with CZP, especially in first line. After two years of treatment, Heinonen and al. did not find any significant differences of retention between ADA and ETN prescribed as first bDMARDs, while in our study ADA had significantly better retention compared to ETN ^42^. When 2nd line bDMARDs are considered, no significant differences between treatments were found in our study. A Swedish study focusing on second-line TNFi in axSpA showed significantly higher persistence of GOL than ADA ^43^. Another study from Spain found retention rates of 80% at 1 year and 70% at 2 years for GOL prescribed as second-line bDMARDs ^44^.

In our population, ETN was the best retained SC-TNFi only when prescribed in at least 3rd line of treatment, while it had previously been reported as a well-maintained SC-TNFi with equivalent or even superior retention compared with other TNFis ^13,37^. In an open label extension phase of randomized clinical trials, reported rates of drug survival were 76% at 96 weeks ^45^. An Austrian study showed a survival rate of 83% at 1 year for ETN ^46^. Similarly, another study found 51% of maintenance after 7 years of ETN in axSpA patients, which is pronouncedly higher than in our study, where median survival of ETN was 22 months ^47^. No explanation for this lower retention rate in our study was found since its prescription was associated with neither a particular patient profile, nor with distinguishable reasons for cessation.

In our study, treatment retention was higher in men. Sex was also a predictive factor for treatment cessation in the multivariable analysis. This point is well-described in the literature and stands as a well-known feature of axSpA treatments (either TNFi^5,11,13,28,33,34,36,37,48–51^ or IL-17 inhibitors^52^). Some studies have not found comparable influence of gender, but they are less numerous^12^. It is now widely recognized that women suffering from axSpA have higher disease burden with more severe patient-reported symptoms, as recently confirmed in the US CORRONA registry^53^. This point is likely to affect treatment retention. Treatment inefficiency as the most frequent reason for treatment cessation in our population is likewise concordant.

Among others and as found in our study, Flouri et al. found an association between less retention of treatments and presence of peripheral disease^13,48^. However, Kristensen et al. found presence of peripheral disease as a predictive factor for better treatment retention^5^. In their study, follow-up was limited to 2 years, while in studies demonstrating a negative impact of peripheral disease of treatment retention, follow-up was longer, which could explain, among other hidden factors, the discrepancy between these results. Moreover, women with poorer treatment retention more frequently present with peripheral disease^53^.

To note, while HLA-B27 positivity was associated with better treatment retention in univariable analysis, this factor was not significant in the multivariable analysis of predictive factors for treatment cessation. Our result is concordant with previous reports ^11,12,54^.

In terms for side effects, there was no particular tolerance signal in our study.

Our study had some limitations. First, analysis of some crucial points reported in the literature as influencing treatment retention (smoking, comorbidity score…) was not possible ^10^. Distinguishing Ankylosing Spondylitis (i.e. r-axSpA) from nr-axSpA with certainty was limited due to missing data. Limitation due to missing data also applies to the presence of extra-articular symptoms and diagnostic delay. Indeed, when patients had positive MRI, they did not always receive X-rays. However, there is some evidence in the literature that there is no difference in therapeutic maintenance between nr-axSpA and r-axSpA ^55,56^, which means that this point is not likely to affect our results. Of note, it is not consensual ^54,57^. Another limitation is the absence of information about NSAID consumption while being treated with bDMARDs. Indeed, since patients often consume NSAID only a few times a year, it was not possible to capture this information precisely in our database. This may also explain why this information is lacking in numerous studies focusing on retention rate of bDMARDs in rheumatic diseases^58,59^.

In conclusion, in our multicentre study, GOL showed a significantly higher retention rate in axSpA, with a mean retention length of 59 months. ETN had the best retention rate when prescribed as at least 3rd line bDMARDs. Male sex, absence of peripheral disease, and early line of prescription are associated with better SC-TNFi retention in axSpA. Each treatment had particular predictive factors for retention. Tailoring and prioritizing bDMARD prescription in axSpA could lead to improved patient management.

### Supplementary Information

Supplementary Information.

## Data Availability

The data presented in this study are available on request from the corresponding author.

## References

[1] Navarro-Compán V, Sepriano A, El-Zorkany B, van der Heijde D (2021). Axial spondyloarthritis. Ann. Rheum. Dis..

[2] van der Heijde D (2017). 2016 update of the ASAS-EULAR management recommendations for axial spondyloarthritis. Ann. Rheum. Dis..

[3] Sepriano A (2017). Efficacy and safety of biological and targeted-synthetic DMARDs: A systematic literature review informing the 2016 update of the ASAS/EULAR recommendations for the management of axial spondyloarthritis. RMD Open.

[4] Svedbom A, Storck C, Kachroo S, Govoni M, Khalifa A (2017). Persistence with golimumab in immune-mediated rheumatic diseases: a systematic review of real-world evidence in rheumatoid arthritis, axial spondyloarthritis, and psoriatic arthritis. Patient Prefer. Adherence.

[5] Kristensen LE (2010). Presence of peripheral arthritis and male sex predicting continuation of anti-tumor necrosis factor therapy in ankylosing spondylitis: An observational prospective cohort study from the South Swedish arthritis treatment group register. Arthritis Care Res..

[6] Carmona L, Gómez-Reino JJ (2006). Survival of TNF antagonists in spondylarthritis is better than in rheumatoid arthritis. Data from the Spanish registry BIOBADASER. Arthritis Res. Ther..

[7] Lie E (2011). Effectiveness of switching between TNF inhibitors in ankylosing spondylitis: data from the NOR-DMARD register. Ann. Rheum. Dis..

[8] Conti F (2007). Switching tumour necrosis factor antagonists in patients with ankylosing spondylitis and psoriatic arthritis: an observational study over a 5-year period. Ann. Rheum. Dis..

[9] Hebeisen M (2019). Comparison of drug survival on adalimumab, etanercept, golimumab and infliximab in patients with axial spondyloarthritis. PLOS One.

[10] Iannone F (2018). Influence of baseline modified Rheumatic Disease Comorbidity Index (mRDCI) on drug survival and effectiveness of biological treatment in patients affected with Rheumatoid arthritis, Spondyloarthritis and Psoriatic arthritis in real-world settings. Eur. J. Clin. Invest..

[11] Barata C, Rodrigues AM, Canhão H, Vinga S, Carvalho AM (2021). Predicting biologic therapy outcome of patients with spondyloarthritis: Joint models for longitudinal and survival analysis. JMIR Med. Inform..

[12] Yahya F (2018). Tumour necrosis factor inhibitor survival and predictors of response in axial spondyloarthritis—Findings from a United Kingdom cohort. Rheumatology.

[13] Kang J-H (2014). Drug survival rates of tumor necrosis factor inhibitors in patients with rheumatoid arthritis and ankylosing spondylitis. J. Korean Med. Sci..

[14] Monti S, Grosso V, Todoerti M, Caporali R (2018). Randomized controlled trials and real-world data: Differences and similarities to untangle literature data. Rheumatology.

[15] Salmon J-H (2020). Actual persistence of abatacept in rheumatoid arthritis: results of the french-Ric network. J. Clin. Med..

[16] Larid G (2022). Differential retention of adalimumab and etanercept biosimilars compared to originator treatments: Results of a retrospective French multicenter study. Front. Med..

[17] Rudwaleit M (2009). The development of Assessment of SpondyloArthritis international Society classification criteria for axial spondyloarthritis (part II): Validation and final selection. Ann. Rheum. Dis..

[18] Linden SVD, Valkenburg HA, Cats A (1984). Evaluation of diagnostic criteria for ankylosing spondylitis. Arthritis Rheum..

[19] Rudwaleit M (2009). Defining active sacroiliitis on magnetic resonance imaging (MRI) for classification of axial spondyloarthritis: A consensual approach by the ASAS/OMERACT MRI group. Ann. Rheum. Dis..

[20] Manara M (2017). Two-year retention rate of golimumab in rheumatoid arthritis, psoriatic arthritis and ankylosing spondylitis: Data from the LORHEN registry. Clin. Exp. Rheumatol..

[21] Thomas K (2018). High 3-year golimumab survival in patients with rheumatoid arthritis, ankylosing spondylitis and psoriatic arthritis: real world data from 328 patients. Clin. Exp. Rheumatol..

[22] Goupille P (2022). Real-life golimumab persitence in patients with axial spondyloarthritis: post-hoc results of the prospective observational cohort study, GO-PRACTICE. Clin. Exp. Rheumatol..

[23] Luttropp K (2019). Real-world treatment persistence of golimumab in the management of immune-mediated rheumatic diseases in Europe: A systematic literature review. BMJ Open.

[24] Rahman P (2020). Long-term effectiveness and safety of infliximab and golimumab in ankylosing spondylitis patients from a Canadian prospective observational registry. BMC Rheumatol..

[25] Iannone F (2017). Golimumab in real-life settings: 2 Years drug survival and predictors of clinical outcomes in rheumatoid arthritis, spondyloarthritis, and psoriatic arthritis. Semin. Arthritis Rheum..

[26] Schulze-Koops H (2015). Factors influencing the patient evaluation of injection experience with the SmartJect autoinjector in rheumatoid arthritis. Clin. Exp. Rheumatol..

[27] Calvo-Alén J (2017). Non-adherence to subcutaneous biological medication in patients with rheumatoid arthritis: a multicentre, non-interventional study. Clin. Exp. Rheumatol..

[28] Glintborg B (2010). Predictors of treatment response and drug continuation in 842 patients with ankylosing spondylitis treated with anti-tumour necrosis factor: results from 8 years’ surveillance in the Danish nationwide DANBIO registry. Ann. Rheum. Dis..

[29] Favalli EG (2017). Eight-year retention rate of first-line tumor necrosis factor inhibitors in spondyloarthritis: A multicenter retrospective analysis—Survival of TNF inhibitors over eight years in SpA. Arthritis Care Res..

[30] Yu C-L, Yang C-H, Chi C-C (2020). Drug survival of biologics in treating ankylosing spondylitis: A systematic review and meta-analysis of real-world evidence. BioDrugs.

[31] Tymms K (2018). Treatment patterns among patients with rheumatic disease (rheumatoid arthritis (RA), ankylosing spondylitis (AS), psoriatic arthritis (PsA) and undifferentiated arthritis (UnA)) treated with subcutaneous TNF inhibitors. Clin. Rheumatol..

[32] García-Lagunar MH (2017). Reasons for discontinuation and adverse effects of TNFα inhibitors in a cohort of patients with rheumatoid arthritis and ankylosing spondylitis. Ann. Pharmacother..

[33] Heiberg MS (2008). The comparative one-year performance of anti–tumor necrosis factor α drugs in patients with rheumatoid arthritis, psoriatic arthritis, and ankylosing spondylitis: Results from a longitudinal, observational, multicenter study. Arthritis Rheum..

[34] Brocq O (2007). Maintien thérapeutique des trois anti-TNF disponibles, dans la polyarthrite rhumatoïde (PR), la spondylarthrite ankylosante (SA) et le rhumatisme psoriasique (RP): À propos de 571 prescriptions d’anti-TNF chez 442 patients sur une période de six ans. Rev. Rhum..

[35] Chiowchanwisawakit P (2019). Effectiveness and drug survival of anti-tumor necrosis factor α therapies in patients with spondyloarthritis: Analysis from the thai rheumatic disease prior authorization registry. JCR J. Clin. Rheumatol..

[36] Glintborg B (2013). Clinical response, drug survival and predictors thereof in 432 ankylosing spondylitis patients after switching tumour necrosis factor α inhibitor therapy: Results from the Danish nationwide DANBIO registry. Ann. Rheum. Dis..

[37] Jeong H (2018). Drug survival of tumor necrosis factor α inhibitors in patients with ankylosing spondylitis in Korea. Korean J. Intern. Med..

[38] Krajewski F, Andras L, Pereira-Gillion C, Goupille P, Salliot C (2019). Drug maintenance of a second tumor necrosis factor alpha inhibitor in spondyloarthritis patients: A real-life multicenter study. J.t Bone Spine.

[39] on behalf of the Rheumatologists of Swiss Clinical Quality Management Program for Axial Spondyloarthritis *et al.* Does the reason for discontinuation of a first TNF inhibitor influence the effectiveness of a second TNF inhibitor in axial spondyloarthritis? Results from the Swiss Clinical Quality Management Cohort. *Arthritis Res. Ther.* **18**, 71 (2016). 10.1186/s13075-016-0969-2 PMC4802885 27000865

[40] Rosales-Alexander JL, Balsalobre Aznar J, Pérez-Vicente S, Magro-Checa C (2015). Drug survival of anti-tumour necrosis factor α therapy in spondyloarthropathies: Results from the Spanish emAR II Study. Rheumatology.

[41] Iannone F (2019). Effectiveness of Certolizumab-Pegol in rheumatoid arthritis, spondyloarthritis, and psoriatic arthritis based on the BIOPURE registry: Can early response predict late outcomes?. Clin. Drug Investig..

[42] Heinonen AV (2015). Effectiveness and drug survival of TNF inhibitors in the treatment of ankylosing spondylitis: A prospective cohort study. J. Rheumatol..

[43] Dalén J, Svedbom A, Black CM, Kachroo S (2017). Second-line treatment persistence and costs among patients with immune-mediated rheumatic diseases treated with subcutaneous TNF-alpha inhibitors. Rheumatol. Int..

[44] Alegre-Sancho JJ (2021). Effectiveness and persistence of golimumab as a second biological drug in patients with spondyloarthritis: A retrospective study. Medicine.

[45] Davis JC (2005). Sustained durability and tolerability of etanercept in ankylosing spondylitis for 96 weeks. Ann. Rheum. Dis..

[46] Nell-Duxneuner V, Schroeder Y, Reichardt B, Bucsics A (2012). The use of TNF-inhibitors in ankylosing spondylitis in Austria from 2007 to 2009—A retrospective analysis. Int. J. Clin. Pharmacol. Ther..

[47] Arends S (2017). Long-term drug survival and clinical effectiveness of etanercept treatment in patients with ankylosing spondylitis in daily clinical practice. Clin. Exp. Rheumatol..

[48] Flouri ID (2018). Comparative analysis and predictors of 10-year tumor necrosis factor inhibitors drug survival in patients with spondyloarthritis: First-year response predicts longterm drug persistence. J. Rheumatol..

[49] Lubrano E (2018). The sex influence on response to tumor necrosis factor-α inhibitors and remission in axial spondyloarthritis. J. Rheumatol..

[50] Fafá BP (2015). Drug survival and causes of discontinuation of the first anti-TNF in ankylosing spondylitis compared with rheumatoid arthritis: Analysis from BIOBADABRASIL. Clin. Rheumatol..

[51] Rusman T (2018). Gender differences in retention rate of tumor necrosis factor alpha inhibitor treatment in ankylosing spondylitis: A retrospective cohort study in daily practice. Int. J. Rheum. Dis..

[52] Alonso S (2021). Multicenter study of secukinumab survival and safety in spondyloarthritis and psoriatic arthritis: Secukinumab in cantabria and ASTURias study. Front. Med..

[53] Mease PJ (2021). Comparison of Men and women with axial spondyloarthritis in the US-based corrona psoriatic arthritis/spondyloarthritis registry. J. Rheumatol..

[54] Lopalco G (2019). Different drug survival of first line tumour necrosis factor inhibitors in radiographic and non-radiographic axial spondyloarthritis: A multicentre retrospective survey. Clin. Exp. Rheumatol..

[55] Michelena X (2021). Similar biologic drug response regardless of radiographic status in axial spondyloarthritis: Data from the British society for rheumatology biologics register in ankylosing spondylitis registry. Rheumatology.

[56] Corli J (2015). Tumor necrosis factor-α inhibition in ankylosing spondylitis and nonradiographic axial spondyloarthritis: Treatment response, drug survival, and patient outcome. J. Rheumatol..

[57] Praprotnik S, Tomsic M (2020). Comment on: Different drug survival of first line tumour necrosis factor inhibitors in radiographic and non-radiographic axial spondyloarthritis—A multicentre retrospective survey. Clin. Exp. Rheumatol..

[58] Dougados M (2023). Factors associated with the retention of secukinumab in patients with axial spondyloarthritis in real-world practice: Results from a retrospective study (FORSYA). RMD Open.

[59] Lie E (2015). The effect of comedication with conventional synthetic disease modifying antirheumatic drugs on TNF inhibitor drug survival in patients with ankylosing spondylitis and undifferentiated spondyloarthritis: Results from a nationwide prospective study. Ann. Rheum. Dis..

