# Supplementary material for: Retention rate of subcutaneous TNF inhibitors in axial spondyloarthritis in a multicentre study from the RIC-FRANCE network
Source: Sci Rep. 2024 Jan 16;14:1374. doi: 10.1038/s41598-024-52016-4 (PMC10791989; doi:10.1038/s41598-024-52016-4)
Supplement: Supplementary file 1 — Supplementary Information. [file 41598_2024_52016_MOESM1_ESM.docx]

Supplementary Figure 1: Retention rate of treatments depending on the line of prescription


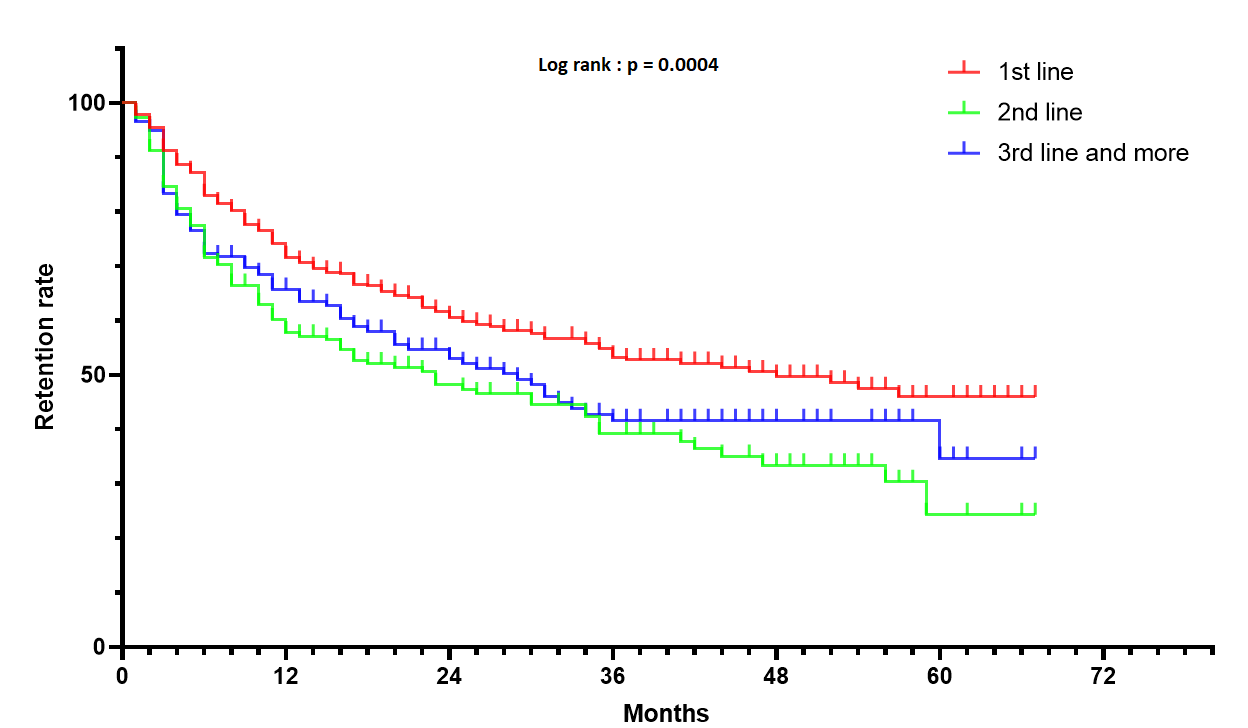


Comparison of retention curves with a log-rank test using Kaplan Meier curves

Supplementary Figure 2: Retention rate of SC-TNFi depending on the sex


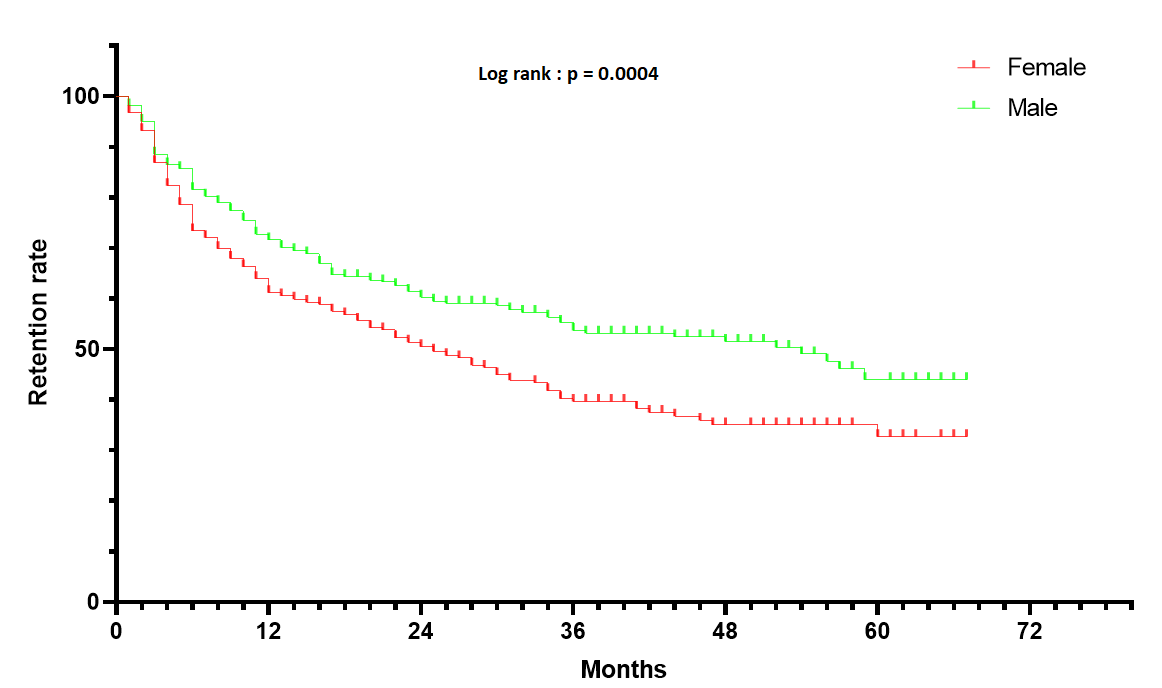


Comparison of retention curves with a log-rank test using Kaplan Meier curves

Supplementary Figure 3: Retention rate of ETN and Monoclonal Antibodies (Mab) in men (A) and women (B)


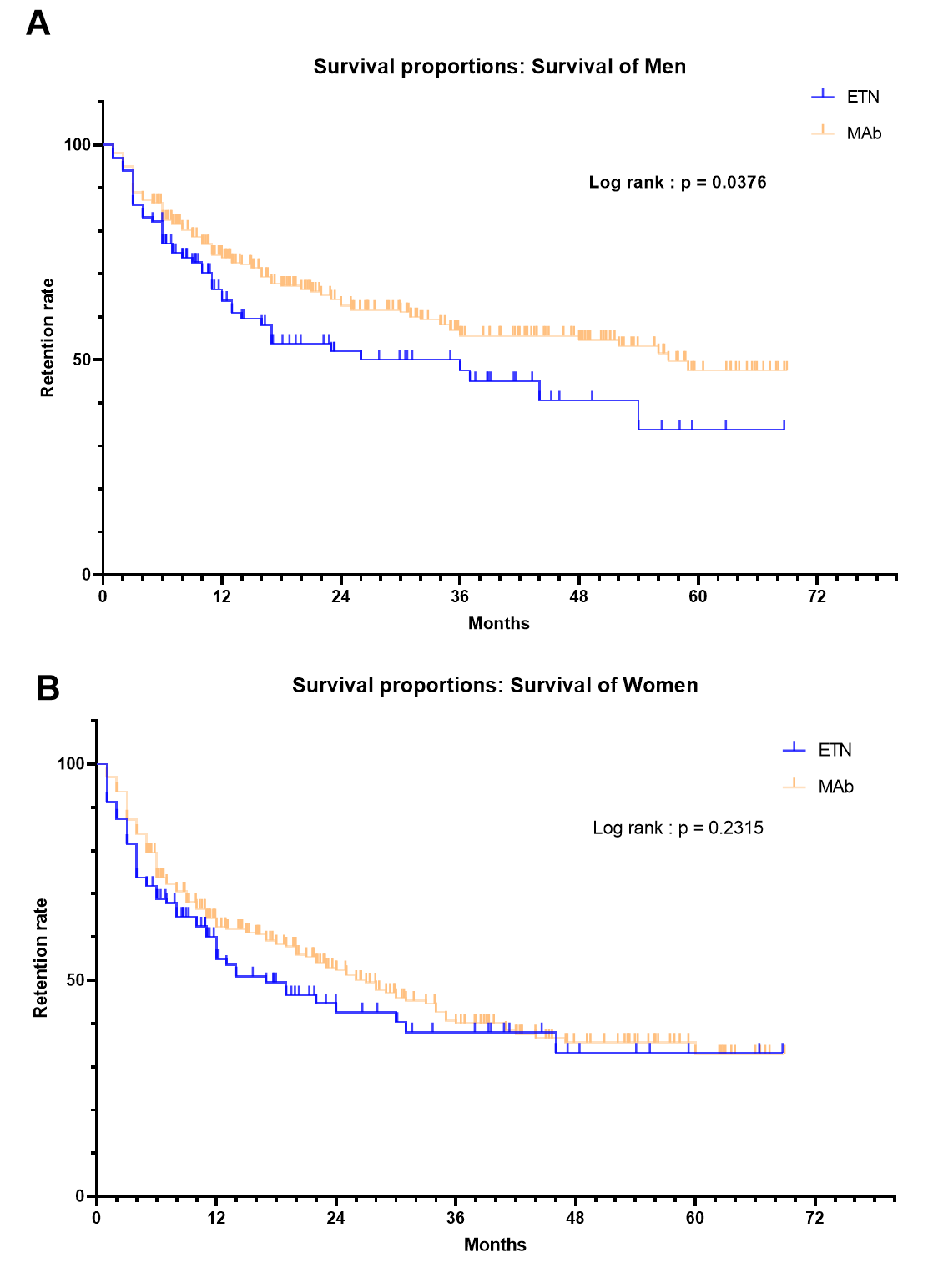


Comparison of retention curves with a log-rank test using Kaplan Meier curves; ETN = etanercept; Mab = monoclonal antibodies (golimumab + adalimumab + certolizumab pegol)

Supplementary Figure 4: Retention rate of SC-TNFi depending on the HLA-B27 positivity


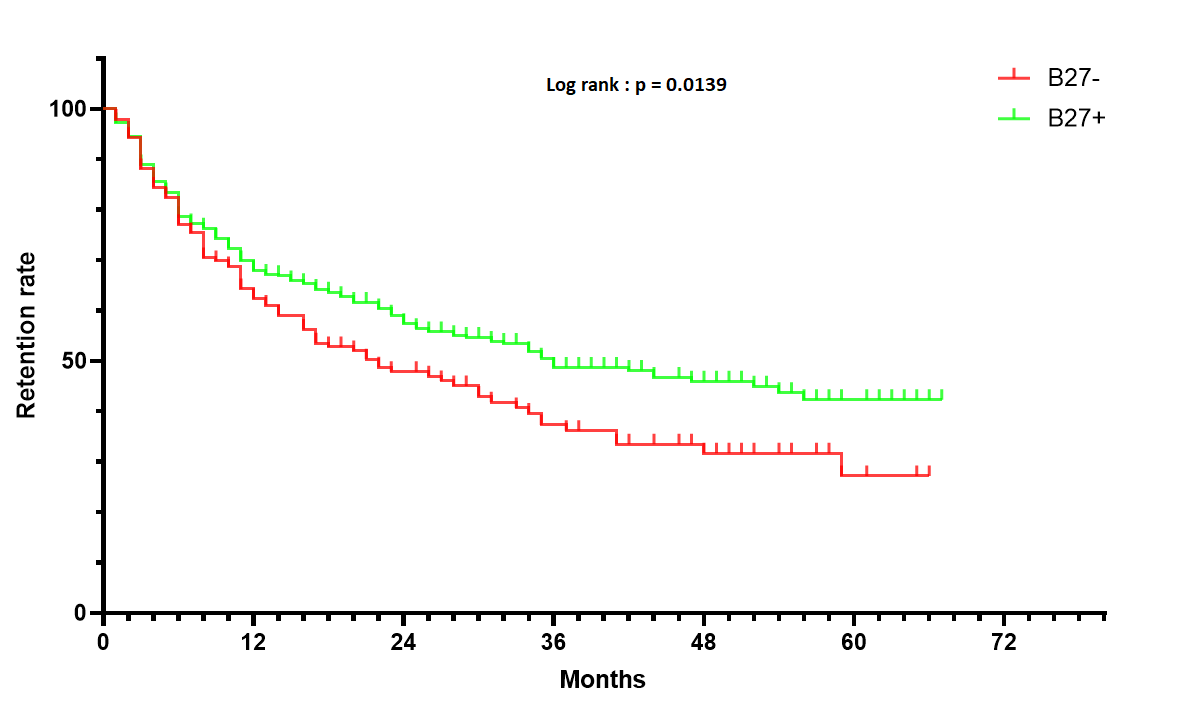


Comparison of retention curves with a log-rank test using Kaplan Meier curves

Supplementary Table 1: Comparison of prescription differences depending on the line of treatment

|  | CZP | GOL | ETN |
| --- | --- | --- | --- |
| 1^st^ line |  |  |  |
| ADA | **OR = 20.98**  **(11.42-39.01)**  **p < 0.0001** | **OR = 1.437**  **(1.077-1.905)**  **p = 0.0132** | **OR = 1.540**  **(1.150-2.051)**  **p = 0.0034** |
| ETN | **OR = 13.62**  **(7.5444-25.55)**  **p < 0.0001** | 0.9332  (0.6907-1.259)  p = 0.6488 | - |
| GOL | **OR = 14.60**  **(8.104-27.33)**  **p < 0.0001** | - | - |
| 2^nd^ line |  |  |  |
| ADA | **OR = 8.658**  **(5.229-14.50)**  **p < 0.0001** | **OR = 4.010**  **(2.613-6.119)**  **p < 0.0001** | **OR = 2.410**  **(1.632-3.551)**  **p < 0.0001** |
| ETN | **OR = 3.592**  **(2.077-6.200)**  **p < 0.0001** | **OR = 1.664**  **(1.073-2.632)**  **p = 0.0248** | - |
| GOL | **OR = 2.159**  **(1.252-3.723)**  **p = 0.0063** | - | - |
| 3rd line |  |  |  |
| ADA | **OR = 0.5275**  **(0.3053-0.9005)**  **p = 0.0174** | **OR = 0.2225**  **(0.1344-0.3676)**  **p < 0.0001** | OR = 1.094  (0.6042-2.005)  p = 0.7639 |
| ETN | **OR = 0.4820**  **(0.2840-0.8364)**  **p = 0.0076** | **OR = 0.2033**  **(0.1192-0.3417)**  **p < 0.0001** | - |
| GOL | **OR = 2.371**  **(1.524-3.663)**  **p = 0.0001** | - | - |

Significant values are in bold. p values < 0.05 are considered as significant. Odd ratios computed using the Baptista-Pike method representing line vs column analysis. p < 0.05 considered significant. ETN : etanercept, ADA : adalimumab, CZP : certolizumab pegol, GOL : golimumab
